# Cranial Base Morphology and Mandibular Growth in Skeletal Deep Bite: A Longitudinal Study in Prepubertal Children

**DOI:** 10.3390/diagnostics16101414

**Published:** 2026-05-07

**Authors:** Eugen Silviu Bud, Mariana Păcurar, Cristina Ioana Bica, Sorana Maria Bucur, Alexandru Vlasa, Beáta Szabó, Anamaria Bud

**Affiliations:** 1 Orthodontics Department, Faculty of Dental Medicine, George Emil Palade University of Medicine, Pharmacy, Science and Technology of Târgu Mureș, 540142 Târgu Mureș, Romania; eugen.bud@umfst.ro (E.S.B.); mariana.pacurar@umfst.ro (M.P.); 2 Pedodontics Department, Faculty of Dental Medicine, George Emil Palade University of Medicine, Pharmacy, Science and Technology of Târgu Mureș, 540142 Târgu Mureș, Romania; cristina.bica@umfst.ro (C.I.B.); anamaria.bud@umfst.ro (A.B.); 3 Department of Dentistry, Faculty of Medicine, “Dimitrie Cantemir” University of Târgu Mureș, 540545 Târgu Mureș, Romania; 4 Department of Periodontology and Oral-Dental Diagnosis, Faculty of Dental Medicine, George Emil Palade University of Medicine, Pharmacy, Science and Technology of Târgu Mureș, 38 Gh. Marinescu Str., 540142 Târgu Mureș, Romania; 5 Private Office, 14 Ion Heliade Rădulescu Str., 540189 Târgu Mureș, Romania; szabo.beata05@gmail.com

**Keywords:** skeletal deep bite, cranial base morphology, hypodivergent growth pattern, mandibular rotation, cephalometric analysis, longitudinal craniofacial growth

## Abstract

**Background:** Skeletal deep bite malocclusion (Angle Class II division 2) represents a common vertical skeletal discrepancy characterized by excessive anterior overbite and a hypodivergent craniofacial growth pattern. The contribution of cranial base morphology to mandibular growth direction during early development remains incompletely understood. **Objective:** To evaluate the relationship between cranial base morphology and maxillofacial growth patterns in children with skeletal deep bite, using a longitudinal cephalometric approach. **Materials and Methods:** This retrospective longitudinal cohort study included 96 prepubertal patients aged 7–10 years (54 with skeletal deep bite, 42 Angle Class I dentoalveolar controls). Skeletal deep bite was defined by overbite > 4 mm and mandibular plane angle (FMA) < 22°. A total of 298 standardized lateral cephalometric radiographs (2–4 per subject; mean follow-up 24 ± 4 months) were analyzed. Twenty-two cephalometric parameters were assessed. Longitudinal changes were evaluated using repeated-measures analysis, and predictors of deep bite severity were identified using multiple linear regression. **Results:** Compared with controls, the deep bite group exhibited significantly reduced mandibular plane angle (18.5° vs. 24.0°), smaller gonial angle (104.3° vs. 111.0°), and decreased lower anterior facial height (86.3 mm vs. 92.0 mm; all *p* < 0.001). Differences in cranial base morphology were modest, including reduced anterior cranial base length (58.6 mm vs. 60.0 mm; *p* = 0.031). Regression analysis identified gonial angle and lower anterior facial height as significant predictors (R^2^ = 0.58). Longitudinal analysis suggested early tendencies toward forward mandibular rotation and reduced vertical growth rate over the observation period. **Conclusions:** In prepubertal children, skeletal deep bite is associated with early tendencies toward reduced vertical facial development and forward mandibular rotation. Cranial base morphology appears to be associated with mandibular growth direction as a secondary modulatory factor rather than a primary determinant. Early identification of hypodivergent growth indicators may facilitate timely interceptive orthodontic strategies.

## 1. Introduction

**Figure 1:**
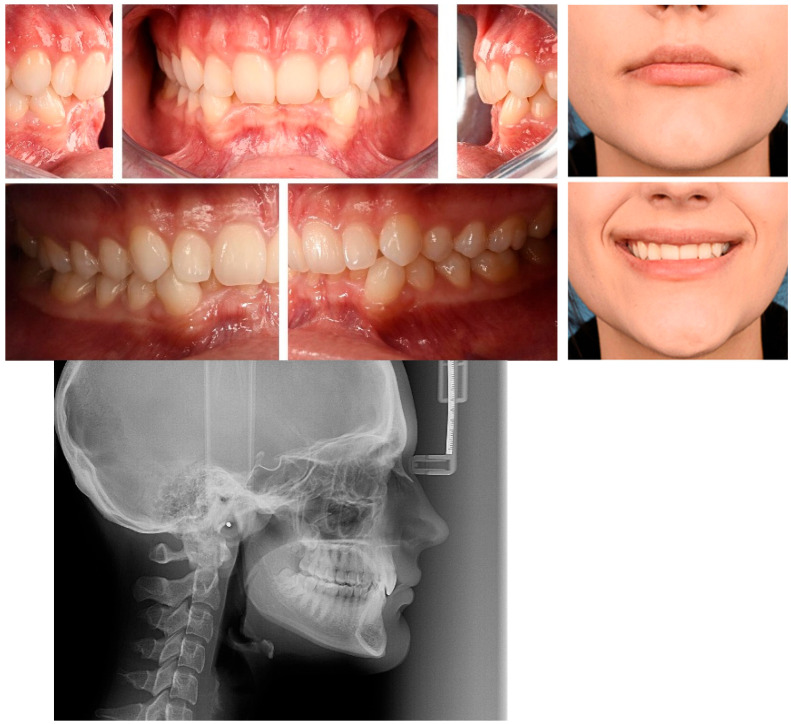
Skeletal deep bite malocclusion, oral aspects, and lateral cephalometry.

Skeletal deep bite malocclusion (Angle Class II division 2) is a complex dento-maxillary anomaly characterized by excessive vertical overlap of the anterior teeth, frequently associated with skeletal, dentoalveolar, and functional disturbances [1,2] (Figure 1). It represents one of the most common vertical malocclusions encountered in orthodontic practice and has important implications for craniofacial growth, occlusal function, and long-term treatment stability.

Epidemiological studies estimate a worldwide prevalence of approximately 21%, with reported values ranging from 8% to 51% depending on diagnostic criteria, age group, and population characteristics. This condition affects nearly one-fifth of children and approximately 13% of adults, highlighting its clinical relevance, particularly during active growth phases [1,2,3].

The etiopathogenesis of skeletal deep bite is multifactorial, involving interactions among cranial base morphology, mandibular growth direction, vertical facial development, and genetic influences [1,2,3,4]. Among these, genetic factors play a fundamental role in regulating craniofacial growth patterns, while environmental and functional factors may further modulate their expression [5]. Despite its high prevalence, the developmental mechanisms underlying skeletal deep bite remain incompletely understood, particularly in relation to early growth dynamics.

The cranial base plays a central role in craniofacial development by providing structural support and spatial orientation for the maxillofacial complex [6,7]. Variations in cranial base morphology, including differences in length and angulation, may influence sagittal and vertical skeletal relationships [8]. Altered growth patterns at cranial base synchondroses have been suggested to contribute to dento-maxillary anomalies by modifying maxillary and mandibular positioning through changes in cranial base flexure [8,9,10,11,12,13,14].

Although numerous studies have investigated the relationship between cranial base morphology and skeletal malocclusions, their findings remain heterogeneous, reflecting differences in study design, age groups, and cephalometric methodologies [10,11,12]. Most available data are derived from cross-sectional studies or from adolescent and adult populations, limiting the understanding of early developmental processes.

Despite existing evidence on cranial base morphology and mandibular growth, relatively few longitudinal studies have simultaneously evaluated these relationships during the prepubertal period. Consequently, the interaction between cranial base configuration and mandibular growth direction during early mixed dentition remains insufficiently clarified [9,10,11]. This limitation is particularly relevant, as etiopathogenic factors are expected to exert their strongest influence during early craniofacial development [2,4,6].

Therefore, this study aimed to longitudinally evaluate the relationship between cranial base morphology and mandibular growth direction in prepubertal children with skeletal deep bite compared to Angle Class I controls. Additionally, the study aimed to identify early predictors of hypodivergent growth patterns and to assess growth tendencies during a critical developmental period preceding pubertal acceleration.

## 2. Materials and Methods

### 2.1. Ethical Approval

This retrospective longitudinal cohort study was conducted using records of the Department of Orthodontics at the University of Medicine, Pharmacy, Science and Technology “George Emil Palade” of Târgu Mureș, collected between January 2022 and December 2024. Ethical approval was obtained from the institutional University’s Ethics Committee (Approval No. 2709/27 December 2020), and written informed consent from the parents or legal guardians of all participants was obtained before inclusion. All radiographs were part of routine clinical care; no imaging was performed solely for research purposes.

### 2.2. Study Design and Population

This retrospective longitudinal cohort study followed the STROBE guidelines for observational studies to evaluate craniofacial growth patterns in pediatric patients undergoing orthodontic assessment. The sample consisted of 96 subjects in active growth stages who were monitored between 2022 and 2024 at the Orthodontics and Dentofacial Orthopedics Clinic.

Patient selection followed a consecutive sampling framework. The clinical database of the Orthodontics and Dentofacial Orthopedics Clinic was screened for all patients aged 7–10 years who underwent baseline orthodontic assessment between January 2022 and December 2024. Subjects meeting the predefined inclusion criteria were consecutively enrolled until the target sample size was reached. No additional matching or selective recruitment procedures were applied. This approach minimized selection bias by including all eligible patients within the defined time interval.

The longitudinal design enabled serial evaluation of craniofacial development using standardized lateral cephalometric radiographs obtained at multiple time points during growth. In total, 298 radiographs were analyzed. All radiographs were obtained in centric occlusion.

Based on clinical examination and cephalometric diagnosis, subjects were allocated into two groups:

(1) Study group: 54 patients presenting skeletal deep bite malocclusion (Angle Class II division 2);
(2) Control group: 42 patients presenting Angle Class I dentoalveolar malocclusions without skeletal sagittal discrepancies. Angle Class I dentoalveolar malocclusion without skeletal discrepancy was used as a control group, representing near-normal skeletal growth patterns. This approach has been used in previous cephalometric growth studies to balance ethical considerations and methodological comparability.

Exclusion criteria included genetic syndromes, metabolic disorders, craniofacial malformations, systemic growth disturbances, and previous orthodontic treatment.

Growth stage standardization was approximated using chronological age at baseline, with all subjects included between 7 and 10 years, corresponding predominantly to pre-pubertal developmental phases. Cervical vertebral maturation (CVM) stages were not systematically recorded due to the retrospective design. Consequently, inter-individual variability in skeletal maturation within the 7–10-year age range cannot be fully excluded. Although this interval predominantly corresponds to pre-pubertal growth phases, differences in biological maturation may have influenced individual growth trajectories. Therefore, the findings should be interpreted as reflecting general early growth tendencies rather than stage-specific skeletal development. However, the narrow age interval and longitudinal design reduced variability in skeletal maturation between groups [15,16,17]. Although the longitudinal design allowed repeated observations, the observation period (approximately 24 months) reflects early growth monitoring and does not encompass the full pubertal growth phase.

It is recognized that using Angle Class I dentoalveolar malocclusion as a control group does not fully reflect ideal craniofacial development, as minor skeletal discrepancies may still be present. These subtle variations can diminish the observed differences between groups; consequently, this cohort should be regarded as representing near-normal rather than optimal growth patterns [10]. Accordingly, the reported findings are likely to represent conservative estimates of the true deviation from ideal craniofacial norms.

### 2.3. Cephalometric Analysis

Each participant underwent between two and four lateral cephalometric radiographic examinations during the observation period. Radiographs were obtained exclusively for clinical diagnostic and monitoring purposes, in accordance with standard orthodontic protocols, and no additional radiographs were taken for research reasons.

All lateral cephalometric radiographs were obtained using the same digital cephalometric unit (Planmeca ProMax^®^, Helsinki, Finland) with a standardized magnification factor of 1.1. Images were exported in calibrated digital format, and measurements were performed using Planmeca Romexis^®^ software (version 6.2.0), which incorporates automatic magnification correction. Head positioning was standardized using cephalostat ear rods and a fixed nasion support. Patients were instructed to maintain natural head position with teeth in centric occlusion and lips relaxed. For longitudinal comparisons, reference structures of the cranial base were used to minimize the influence of minor positioning variations across repeated radiographs.

The timing of radiographic acquisition was determined by clinical follow-up needs, typically at baseline and at approximately 12-month intervals during active growth monitoring. To reduce variability in longitudinal comparisons, repeated-measures statistical models were applied, which account for unequal numbers of observations per patient. Follow-up intervals ranged between 9 and 15 months, reflecting routine clinical scheduling; this range was considered acceptable for longitudinal growth assessment.

Skeletal deep bite was defined by overbite > 4 mm, FMA < 22°, and reduced lower anterior facial height.

These diagnostic thresholds were selected based on previously published pediatric cephalometric norms and studies defining hypodivergent skeletal patterns in mixed dentition. The FMA cut-off of 22° corresponds to the lower limit of normative values reported for growing Caucasian populations, while an overbite greater than 4 mm is widely accepted as a clinically significant deep bite in pediatric orthodontics [2,4,6]. The combined use of skeletal and dental criteria aimed to improve diagnostic specificity for skeletal deep bite during early growth stages.

Cephalometric evaluation included 22 parameters comprising four cranial base measurements and eighteen maxillofacial linear and angular variables. All measurements were performed on digital lateral cephalograms using standardized reference planes and were compared with established normative values for the Caucasian pediatric population.

Definitions of cephalometric landmarks, reference planes, measured variables, and all abbreviations used in the tables are provided in Supplementary Table S1 to facilitate reproducibility of the analysis.

To assess measurement reliability, 30 randomly selected cephalograms were reanalyzed after a two-week interval. Method error was calculated using Dahlberg’s formula, and intra-class correlation coefficients were computed. Method error ranged from 0.3 to 0.8 mm for linear measurements and from 0.4° to 0.9° for angular measurements. Intra-class correlation coefficients ranged between 0.91 and 0.97, indicating excellent intra-examiner reliability.

Because follow-up intervals varied between 9 and 15 months, repeated-measures statistical models were selected to account for unequal observation timing. These models incorporate within-subject variability and do not require uniform time intervals, thereby reducing bias associated with non-standardized follow-up durations. Consequently, growth trends were interpreted as relative changes over time rather than absolute growth rates.

The cranial base measurements included anterior cranial base length (S–N), posterior cranial base length (S–Ba), and cranial base angulations (N–S–Ba and N–Op–Ba), reflecting both linear dimensions and flexure of the cranial base. Key angular measurements included FMA, gonial angle, and facial axis, which characterize the vertical growth pattern and mandibular rotation.

Detailed reliability analysis for each variable is presented in Table 1.

### 2.4. Statistical Analysis

Statistical analyses were conducted using IBM SPSS Statistics (version 29.0; IBM Corp., Armonk, NY, USA). The distribution of variables was assessed with the Shapiro–Wilk test. Descriptive measures comprised means, standard deviations, and 95% confidence intervals.

Between-group differences were evaluated using independent-samples *t*-tests or analysis of variance, as appropriate, while categorical data were analyzed with chi-square tests. Pearson correlation coefficients examined associations between cranial base parameters and maxillofacial measurements. Formal adjustment for multiple comparisons was not performed, given the exploratory nature of the analysis; instead, results were interpreted in light of effect sizes and their biological plausibility, with effect magnitudes quantified using Cohen’s *d*.

Multiple linear regression was performed to identify predictors of skeletal deep bite severity, with FMA defined as the dependent variable. FMA was used as a continuous indicator of vertical skeletal pattern, not as a diagnostic classifier within the regression model. Model adequacy was verified through residual analysis, assessment of multicollinearity, and evaluation of variance inflation factors. Model robustness was further explored using leave-one-out cross-validation.

A priori power analysis indicated that a minimum of 34 subjects per group was required to detect moderate effect sizes with 80% statistical power at a significance level of 0.05. The calculation was based on a moderate effect size (Cohen’s *d* = 0.6) for differences in FMA, selected as a primary outcome variable reflecting vertical skeletal pattern.

Longitudinal data were analyzed by repeated-measures analysis of variance, treating subjects as random effects to account for within-individual correlations from multiple radiographs. A mixed-effects framework with subjects treated as random effects was used to account for repeated observations and unequal numbers of radiographs per subject. This framework accommodated unequal numbers of observations per participant and variable intervals between measurements, enabling reliable estimation of temporal trends. Multicollinearity was assessed using variance inflation factors (VIF), and residual diagnostics were evaluated to confirm model assumptions.

Follow-up duration was additionally examined as a covariate in exploratory analyses and showed no significant influence on primary outcomes, supporting the robustness of the findings despite variability in observation timing.

Statistical significance was set at *p* < 0.05. To address the potential for inflated type I error due to multiple testing, results were interpreted alongside effect sizes and their biological relevance.

## 3. Results

### 3.1. Sample Characteristics and Data Distribution

The longitudinal cohort consisted of 96 patients contributing a total of 298 standardized lateral teleradiographs obtained across multiple observation time points. The unequal distribution between the study and control groups reflected the natural prevalence of the studied malocclusion within the consecutively screened clinical population. The study group included 54 patients presenting the studied skeletal malocclusion features. The control group comprised subjects presenting Angle Class I dento-alveolar malocclusions without skeletal sagittal discrepancies.

Age distribution showed no statistically significant differences between groups (*p* > 0.05), ensuring comparability in growth stage. Sex distribution was balanced.

Before conducting inferential analysis, data normality was assessed using the Shapiro–Wilk test, which confirmed that most variables were normally distributed (*p* > 0.05). Variables that showed mild deviations from normality were examined using skewness and kurtosis and retained, given the robustness of parametric tests for samples exceeding 30 subjects.

The longitudinal nature of the dataset allowed for the evaluation of both structural differences and developmental trends, strengthening the interpretive power of the findings.

### 3.2. Cranial Base Parameters

The cranial base analysis illustrated in Table 2 demonstrated a modest but statistically significant reduction in posterior cranial base angulation in the deep bite group.

The slightly shorter anterior cranial base length observed in the study group is further consistent with the hypothesis that cranial base morphology may influence mandibular spatial orientation by modifying skeletal growth vectors. However, effect sizes remained moderate, indicating that cranial base morphology likely acts as a secondary modulating factor rather than a primary etiological determinant.

### 3.3. Maxillofacial Linear Parameters

Linear measurements (Table 3) demonstrated a consistent pattern of reduced vertical facial dimensions in the deep bite group, particularly in lower anterior facial height (N-Gn and Nsa-Gn), which exhibited large effect sizes. These findings confirm a hypodivergent growth pattern characterized by reduced vertical facial development. Conversely, mandibular body length (Go-Gn) showed a mild increase.

### 3.4. Angular Maxillofacial Parameters

Angular measurements (Table 4) revealed a characteristic hypodivergent skeletal pattern in the deep bite group. The significantly reduced FMA and gonial angles exhibited the largest effect sizes.

The increased facial axis and mandibular prognathism indicators further support the presence of anterior rotational growth.

### 3.5. Regression Analysis

The regression model explained 58% of the variance (R^2^ = 0.58) and identified key predictors:

- Gonial angle (β = −0.41, *p* < 0.001)
- Lower anterior facial height (β = −0.36, *p* = 0.003)

These results indicate that vertical growth parameters are significant predictors of skeletal deep bite severity. Full regression outputs, including coefficients, standard errors, 95% confidence intervals, variance inflation factors, and residual diagnostics, are provided in Table 5.

### 3.6. Longitudinal Growth Trends

Repeated-measures analysis over the observation period indicated:

- Facial height increased in both groups (*p* < 0.001)
- Growth increase was significantly smaller in the deep bite group
- Mandibular plane angle showed a decreasing trend over time, suggesting progressive anterior mandibular rotation.

## 4. Discussion

### 4.1. Study Rationale and Scientific Context

Understanding the determinants of craniofacial growth remains a central challenge in orthodontics, as skeletal malocclusions result from complex interactions among cranial base morphology, mandibular growth direction, vertical facial development, and genetic influences [1,2,5,13,14]. Skeletal deep bite, in particular, is strongly associated with disturbances in vertical growth patterns and presents significant clinical challenges [3,5].

Previous investigations into cranial base morphology have largely relied on cross-sectional designs or focused on adolescent populations, limiting insight into early growth dynamics [15,16,17,18]. Consequently, the temporal relationship between cranial base configuration and mandibular growth direction during early development remains incompletely understood [19].

The present longitudinal study provides data from a prepubertal cohort, enabling evaluation of both structural characteristics and growth trends. The findings indicate that cranial base morphology plays a secondary, modulatory role in mandibular growth direction rather than acting as a primary determinant, which is consistent with previous reports [5,20,21]. This observation aligns with established growth models emphasizing the predominance of mandibular rotational patterns in defining vertical skeletal relationships [15,16,17,18,19]. Similar conclusions have been reported in recent longitudinal imaging studies [22,23].

### 4.2. Interpretation of Major Findings

The results demonstrate a consistent hypodivergent skeletal pattern in the deep bite group, characterized by reduced mandibular plane angle, decreased gonial angle, and diminished lower anterior facial height [3]. These features reflect a horizontal growth tendency associated with forward mandibular rotation [16,17,18].

These findings are consistent with previous studies describing forward mandibular rotation as a defining characteristic of deep bite morphology [15,16,24]. The large effect sizes observed for vertical parameters further support the role of reduced vertical facial development as a key component of deep bite malocclusion [25,26].

Mandibular body length was slightly increased, indicating that a deep bite is more closely related to altered growth direction than to mandibular deficiency [1,27]. Regression analysis identified gonial angle and lower anterior facial height as significant predictors of skeletal deep bite severity, supporting previous multivariate findings on vertical skeletal relationships [2,3,4,27].

Cranial base parameters showed smaller but statistically significant differences, particularly in angulation and anterior length. These findings support the concept that cranial base morphology influences mandibular positioning indirectly, through modulation of growth vectors rather than direct causation [15,17,24,28,29]. However, the magnitude of these differences suggests a limited clinical impact compared with vertical growth parameters.

### 4.3. Significance of Longitudinal Growth Findings

The longitudinal analysis demonstrated that facial height increased in both groups but at a significantly reduced rate in the deep bite cohort. This indicates a relative deficiency in vertical growth rather than a complete absence of growth [30,31].

The observed decrease in mandibular plane angle over time suggests a tendency toward progressive forward mandibular rotation. This pattern is consistent with previous longitudinal studies reporting accentuation of hypodivergent growth during development [1,2,3,4,15,16,17,18,30,31].

These findings highlight the dynamic nature of skeletal deep bite and emphasize the importance of early growth assessment, as treatment opportunities may decrease with advancing skeletal maturation.

### 4.4. Clinical Implications

The identification of reduced lower anterior facial height and forward mandibular rotation as key characteristics has direct clinical relevance. These parameters may serve as early indicators for identifying patients at risk of developing a deep bite.

Early assessment of growth direction during mixed dentition is essential for timely intervention. Interceptive orthodontic approaches aimed at promoting vertical development, including posterior eruption control and bite-opening mechanics, may be beneficial in managing hypodivergent growth patterns [32,33,34,35].

Cranial base assessment may provide additional contextual information in cephalometric analysis; however, its role should be considered secondary to vertical growth parameters [36,37].

### 4.5. Biological and Scientific Implications

The findings support the concept that skeletal deep bite reflects a variation in overall craniofacial growth pattern rather than an isolated dentoalveolar condition [1,2,3,4,30,31]. The predominance of forward mandibular rotation underscores the importance of vertical growth regulation in craniofacial development.

These results are consistent with models emphasizing interactions between genetic factors and functional influences in shaping craniofacial morphology [1,2,3,4]. The secondary contribution of cranial base morphology further supports the integrated nature of craniofacial growth [7,8,9,10,11,12,13,14,19].

Although multiple comparisons were performed, the interpretation of results was guided by effect sizes and biological plausibility. Advances in three-dimensional imaging have provided additional insight into mandibular growth dynamics, particularly regarding condylar remodeling and rotational patterns [38]. Future studies integrating such approaches may further clarify cranial base–mandibular interactions.

Recent literature also supports the clinical relevance of vertical facial parameters in deep bite management, emphasizing the importance of early intervention strategies [6,39].

### 4.6. Limitations and Future Directions

This study has several limitations. The retrospective design introduces potential selection bias, although consecutive sampling was applied. The sample size, while adequate for primary analyses, may limit more complex modeling. The use of Angle Class I malocclusion subjects as controls may not fully represent ideal craniofacial development [10,28,29].

The absence of skeletal maturation indicators, such as CVM staging, limits precise assessment of growth stage variability [15,16,17]. Additionally, the reliance on two-dimensional cephalometry restricts comprehensive spatial evaluation, and the relatively short observation period does not capture pubertal growth changes.

Future longitudinal studies incorporating three-dimensional imaging and biological maturation markers are needed to further elucidate craniofacial growth mechanisms [5,14,27].

## 5. Conclusions

Skeletal deep bite in growing patients is associated with early tendencies toward reduced vertical facial development and forward mandibular rotation. Cranial base morphology appears to be associated with mandibular growth direction as a secondary modulatory factor, although causal relationships cannot be established within the present observational design. Early identification of hypodivergent growth indicators may support timely interceptive orthodontic strategies.

## Figures and Tables

**Table 1:** Intra-examiner reliability analysis (Dahlberg error and ICC).

Parameter	Dahlberg Error	ICC
S–N	0.5 mm	0.94
S–Ba	0.6 mm	0.93
N–S–Ba	0.7°	0.92
N–Op–Ba	0.8°	0.91
FMA	0.6°	0.95
Gonial angle	0.7°	0.94
N–Gn	0.8 mm	0.93

**Table 2:** Cranial Base Measurements.

Parameter	Control (n = 42) Mean	Study (n = 54) Mean	*p*-Value	Cohen’s *d*
∠N-S-Ba (°)	127.8	126.9	>0.05	0.18
∠N-Op-Ba (°)	14.8	13.2	0.018	0.52
S-Ba (mm)	37.3	36.5	>0.05	0.26
S-N (mm)	60.0	58.6	0.031	0.48

**Table 3:** Linear Maxillofacial Measurements.

Parameter	Control (n = 42)	Study (n = 54)	*p*-Value	Effect Size
Nsa-Nsp	42.5	39.8	0.004	0.68
Go-Gn	57.0	59.2	0.028	0.46
S-Nsp	39.0	35.5	<0.001	0.92
N-Nsa	40.5	38.0	0.010	0.62
Nsa-Gn	52.5	47.6	<0.001	1.05
N-Gn	92.0	86.3	<0.001	1.12
Nsp-Go	33.8	32.4	>0.05	0.29
Kdl-Go	40.0	41.5	0.040	0.42

**Table 4:** Angular Measurements.

Parameter	Control (n = 42)	Study (n = 54)	*p*-Value	Effect Size
∠N.S.NsaNsp	6.8	5.4	0.011	0.58
∠N.S-M	31.5	29.2	0.022	0.49
∠N.Nsa-Gn	159.3	165.8	<0.001	1.02
∠S.Nsp-Go	104.8	98.6	<0.001	1.18
∠N.Nsa-Pg	165.0	171.2	<0.001	1.10
∠Go	111.0	104.3	<0.001	1.35
∠SNA	81.3	80.5	>0.05	0.22
∠ANB	3.0	5.2	0.008	0.67
∠FMA	24.0	18.5	<0.001	1.42
∠Nsa.Nsp-M	24.8	21.3	0.004	0.72

**Table 5:** Multiple linear regression analysis identifying predictors of skeletal deep bite severity (FMA as dependent variable).

Predictor	Standardized β	Standard Error	95% Confidence Interval	*p*-Value	VIF
Gonial angle	−0.41	0.09	(−0.58, −0.24)	<0.001	1.6
Lower anterior facial height (N–Gn)	−0.36	0.11	(−0.58, −0.14)	0.003	1.5

Model statistics: R^2^ = 0.58; *p* < 0.001. Diagnostics: Residuals were normally distributed and homoscedastic. No multicollinearity was detected (VIF < 2).

## Data Availability

The original contributions presented in this study are included in the article. Further inquiries can be directed to the corresponding authors.

## Supplementary Materials

The following supporting information can be downloaded at: https://www.mdpi.com/article/10.3390/diagnostics16101414/s1. Supplementary Table S1. Cephalometric landmarks, planes, and measurements used in the study.
