# Supplementary material for: Cranial Base Morphology and Mandibular Growth in Skeletal Deep Bite: A Longitudinal Study in Prepubertal Children
Source: Diagnostics (Basel). 2026 May 7;16(10):1414. doi: 10.3390/diagnostics16101414 (PMC13205141; doi:10.3390/diagnostics16101414)
Supplement: Supplementary file 1 [file diagnostics-4288856-supplementary.pdf › diagnostics-4288856-supplementary.pdf]

REVIEWER 1

Article: Cranial Base Morphology and Mandibular Growth Direction in Skeletal Deep Bite: A Longitudinal Cephalometric Study in Growing Patients

We thank the reviewer for the valuable comments. We believe these revisions have substantially strengthened the manuscript.

Comment 1: The sampling strategy and potential selection bias are unclear.
Response: We agree and have clarified the sampling methodology. A consecutive sampling framework was applied to all eligible patients screened within a defined time interval. The revised text has been added in Section 2.2 (Study Design and Population).

Comment 2: Variable number of radiographs (2–4) requires justification.
Response: Radiographs were obtained strictly for clinical diagnostic purposes, and no additional exposures were performed for research. We clarified the clinical indication, timing, and statistical handling using repeated-measures analysis in Section 2.3.

Comment 3: Were radiographs taken for clinical indication only?
Response: Yes. This has been explicitly stated in Section 2.3, including clarification of follow-up intervals.

Comment 4: Justification for skeletal deep bite cut-offs (FMA <22°, overbite >4 mm).
Response: We added references to pediatric cephalometric norms and clarified diagnostic rationale in Section 2.3.

Comment 5: Control group selection (Class I vs normal occlusion).
Response: We explained ethical limitations regarding radiographic acquisition in normal occlusion subjects and justified the use of Angle Class I dentoalveolar malocclusion as a clinically appropriate comparison group. This has been added to Section 2.2.

Comment 6: Growth stage standardization.
Response: Chronological age standardization and absence of systematic CVM staging were clarified in Section 2.2, and this limitation was acknowledged in the Discussion.

Comment 7: Radiographic equipment and magnification.
Response: The radiographic unit and magnification factor were specified in Section 2.3.

Comment 8: Head positioning reproducibility.
Response: Standardized cephalostat positioning and cranial base reference alignment were described in Section 2.3.

Sincerely,
Sorana Maria Bucur
Corresponding Author

REVIEWER 2

Article: Cranial Base Morphology and Mandibular Growth Direction in Skeletal Deep Bite: A Longitudinal Cephalometric Study in Growing Patients

We thank the reviewer for the valuable comments. We believe these revisions have substantially strengthened the manuscript.

Comment 1: Abstract requires clearer diagnostic criteria and reduced causal claims.
Response: The abstract has been fully revised to include diagnostic thresholds, numerical clarity, and more cautious interpretation.

Comment 2: Research gap not clearly defined.
Response: The introduction now explicitly highlights the lack of longitudinal studies evaluating cranial base influence in early mixed dentition.

Comment 3: Statistical model for repeated measures unclear.
Response: We clarified the use of repeated-measures ANOVA with subjects treated as random effects in Section 2.4.

Comment 4: Cephalometric measurement definitions required.
Response: A Supplementary Table (Table S1) has been added containing definitions of landmarks and variables.

Comment 5: Measurement error values requested.
Response: Method error values and ICC ranges were added in Section 2.3.

Comment 6: Regression model validation.
Response: Residual analysis and cross-validation procedures were described in Section 2.4.

Comment 7: Interpretative wording in Results.
Response: Causal wording has been replaced with associative terminology.

Comment 8: Discussion overemphasizes cranial base role.
Response: The discussion now emphasizes the secondary modulatory effect of cranial base morphology.

Comment 9: Limitations section expansion.
Response: The limitations section has been expanded to include retrospective design, sample size, and imaging constraints.

Comment 10: Inclusion of recent literature.
Response: Additional discussion referencing recent imaging studies has been incorporated.

Sincerely,
Sorana Maria Bucur
Corresponding Author

Cranial Base Morphology and Mandibular Growth Direction in Skeletal Deep Bite: A Longitudinal Cephalometric Study

CONSORT Summary of Study Design and Outcomes

Domain	Summary / Key Details
**Study Type & Focus**	Retrospective longitudinal cephalometric observational study evaluating the relationship between cranial base morphology and maxillofacial growth direction in pediatric patients presenting skeletal deep bite malocclusion (Angle Class II division 2) compared with individuals exhibiting Angle Class I dento-maxillary anomalies.
**Study Population**	Ninety-six growing patients aged 7–10 years were included, comprising 54 subjects with skeletal deep bite malocclusion and 42 controls. A total of 298 standardized lateral cephalometric radiographs were analyzed over a mean longitudinal follow-up period of approximately 24 months.
**Methodology**	Standardized lateral cephalometric radiographs were evaluated at baseline and follow-up. Twenty-two skeletal and cranial base parameters were measured, including anterior and posterior cranial base lengths, cranial base angulation, mandibular plane angle, gonial angle, and vertical facial height indicators. Intergroup comparisons were performed using parametric statistical tests, while longitudinal changes were assessed using repeated-measures analysis and regression modeling.
**Key Quantitative Findings**	The deep bite group demonstrated significantly reduced mandibular plane angle, decreased gonial angle, and diminished lower anterior facial height, indicating a hypodivergent growth pattern ($p < 0.001$). Mandibular body length was slightly increased, reflecting predominant horizontal growth direction. Cranial base morphology showed moderate reductions in posterior cranial base angulation and anterior cranial base length. Regression analysis identified mandibular plane angle and lower facial height as the strongest predictors of deep bite severity ($R^2 \approx 0.58$).
**Primary Clinical Outcomes**	Cranial base morphology parameters; mandibular growth direction indicators; vertical facial height measurements; skeletal predictors of deep bite severity.
**Conclusions & Implications**	Skeletal deep bite malocclusion is primarily determined by a hypodivergent growth pattern characterized by forward mandibular rotation and reduced vertical facial development. Cranial base morphology acts as a secondary modulatory factor influencing mandibular growth direction rather than serving as a primary etiologic determinant.

STROBE Summary of Study Characteristics

Domain	Summary / Key Details
**Study Type & Focus**	Retrospective longitudinal observational cephalometric study assessing relationships between cranial base morphology and skeletal growth pattern in pediatric skeletal deep bite malocclusion.
**Study Population**	Ninety-six growing patients aged 7–10 years, including 54 skeletal deep bite cases and 42 Angle Class I controls, with complete longitudinal radiographic records.

**Methodology**	Cranial base morphology and maxillofacial growth variables were measured using standardized cephalometric analysis. Longitudinal changes were evaluated through repeated-measures statistical modeling.
**Key Quantitative Findings**	Significant hypodivergent skeletal pattern identified in deep bite patients, including reduced mandibular plane angle, decreased gonial angle, diminished lower facial height, and increased horizontal mandibular growth tendency.
**Primary Clinical Outcomes**	Skeletal growth pattern indicators; cranial base angulation and length; predictors of deep bite severity and developmental progression.
**Conclusions & Implications**	Early identification of reduced vertical growth indicators may facilitate timely interceptive orthodontic treatment planning and improve prediction of skeletal deep bite development.

STROBE CHECKLIST

Title and Abstract

STROBE Item	Requirement	Compliance & Comments
**Title**	Indicate study design	Excellent compliance. The title clearly reflects a longitudinal cephalometric observational study.
**Abstract**	Balanced summary	Excellent compliance. Includes objectives, population, methodology, key results, and clinically relevant conclusions.

Introduction

STROBE Item	Requirement	Compliance & Comments
**Background/Rationale**	Explain scientific context	Excellent compliance. Provides epidemiology, etiopathogenesis, and importance of cranial base morphology in deep bite development.
**Objectives**	State clear aims	Excellent compliance. Objectives explicitly address cranial base morphology, growth pattern correlations, and longitudinal analysis.

Methods

STROBE Item	Requirement	Compliance & Comments
**Study Design**	Present early in manuscript	Excellent compliance. Clearly described as retrospective longitudinal observational study.
**Setting**	Describe location/timeframe	Good compliance. Conducted within an academic orthodontic clinical setting with standardized imaging protocols.
**Participants**	Eligibility criteria	Excellent compliance. Inclusion criteria clearly define skeletal deep bite morphology and age range.

**Variables**	Define exposures/outcomes	Excellent compliance. Cranial base morphology as exposure; skeletal growth pattern variables as outcomes.
**Data Sources/Measurement**	Measurement description	Excellent compliance. Standardized cephalometric methodology and reliability testing reported.
**Bias**	Address mitigation	Good compliance. Consecutive sampling and standardized radiographs minimize selection and measurement bias.
**Study Size**	Justify sample size	Excellent compliance. Sample size supported by power analysis.
**Statistical Methods**	Describe analysis	Excellent compliance. Parametric testing, regression modeling, and longitudinal analysis appropriately described.

Results

STROBE Item	Requirement	Compliance & Comments
**Participants**	Report numbers	Excellent compliance. Sample size and group distribution clearly presented.
**Descriptive Data**	Participant characteristics	Excellent compliance. Age, sex distribution, and skeletal parameters summarized.
**Outcome Data**	Report measurements	Excellent compliance. Cranial base and skeletal growth variables comprehensively reported.
**Main Results**	Provide estimates	Excellent compliance. Significant predictors of deep bite severity clearly identified.

Discussion

STROBE Item	Requirement	Compliance & Comments
**Key Results**	Summarize findings	Excellent compliance. Discussion begins with clear synthesis of hypodivergent growth pattern findings.
**Limitations**	Address study limitations	Excellent compliance. Includes retrospective design and two-dimensional imaging limitations.
**Interpretation**	Contextualize findings	Excellent compliance. Results interpreted within existing craniofacial growth literature.
**Funding**	Report funding	Not reported; acceptable if unfunded study.

Supplementary Table S1. Cephalometric landmarks, planes, and measurements used in the study.

Category	Variable	Definition	Unit
Landmark	S (Sella)	Midpoint of sella turcica	—
Landmark	N (Nasion)	Most anterior point of frontonasal suture	—
Landmark	A (Subspinale)	Deepest point on anterior maxillary alveolar contour	—
Landmark	B (Supramentale)	Deepest point on anterior mandibular contour	—
Landmark	Gn (Gnathion)	Most anterior-inferior point of mandibular symphysis	—
Landmark	Go (Gonion)	Most posterior-inferior point of mandibular angle	—
Landmark	Me (Menton)	Lowest point of mandibular symphysis	—
Landmark	ANS	Tip of anterior nasal spine	—
Landmark	PNS	Posterior nasal spine	—
Landmark	Ar (Articulare)	Intersection of posterior ramus and cranial base	—
Landmark	Ba (Basion)	Most inferior posterior point of foramen magnum	—
Plane	SN	Line connecting Sella to Nasion	—
Plane	Mandibular Plane	Line connecting Gonion to Menton	—
Plane	Palatal Plane	Line connecting ANS to PNS	—
Plane	Frankfort Horizontal	Porion to Orbitale	—
Angular	SNA	Maxillary position relative to cranial base	degrees
Angular	SNB	Mandibular position relative to cranial base	degrees
Angular	ANB	Sagittal maxillomandibular relationship	degrees
Angular	FMA	Frankfort horizontal to mandibular plane	degrees
Angular	Gonial Angle	Ar–Go–Me angle	degrees
Angular	SN-GoGn	Mandibular plane inclination to cranial base	degrees
Linear	S–N	Anterior cranial base length	mm
Linear	S–Ba	Posterior cranial base length	mm
Linear	Go–Gn	Mandibular length	mm
Linear	ANS–PNS	Maxillary length	mm
Linear	ANS–Me	Lower anterior facial height	mm
Linear	N–ANS	Upper anterior facial height	mm
Dental	Overbite	Vertical overlap of incisors	mm
Dental	Overjet	Horizontal overlap of incisors	mm

INFORMED CONSENT FORM FOR PARTICIPATION IN RESEARCH (PARENT / LEGAL GUARDIAN)

Study Title:
Cranial Base Morphology and Mandibular Growth Direction in Skeletal Deep Bite: A Longitudinal Cephalometric Study

Principal Investigators:
Eugen Silviu Bud, Mariana Păcurar
Department of Orthodontics, University of Medicine, Pharmacy, Science and Technology "George Emil Palade" of Târgu Mureș
Your child is invited to participate in a clinical research study investigating craniofacial growth patterns in children with orthodontic malocclusions. Participation is entirely voluntary.
The purpose of this study is to evaluate the relationship between cranial base morphology and maxillofacial development during active growth stages in children. The findings aim to improve understanding of the causes, early diagnosis, and treatment planning of skeletal deep bite malocclusion.

Study Procedures
If you agree to allow your child to participate, the following will occur:

- Analysis of existing lateral cephalometric radiographs taken as part of routine orthodontic diagnosis
- Collection of orthodontic measurements from your child's clinical records
- Longitudinal evaluation of craniofacial growth patterns

No additional radiographs, examinations, or procedures beyond standard orthodontic care will be required.

Risks and Discomforts
This study involves minimal risk. All radiographs included are part of routine orthodontic diagnostic procedures and will not require any additional radiation exposure.

Benefits
There is no direct financial or medical benefit to your child from participating in this study. However, the results may contribute to improved orthodontic diagnosis, early treatment planning, and understanding of craniofacial growth patterns in children.

Confidentiality
All collected data will be anonymized. No personal identifying information about your child will appear in publications, reports, or shared materials. Data will be stored securely and accessed only by authorized research personnel.

Voluntary Participation
Participation is voluntary. You may withdraw your child from the study at any time without affecting their medical care or orthodontic treatment.

Ethical Approval
This study has been approved by the Ethics Committee of the University of Medicine, Pharmacy, Science and Technology "George Emil Palade" of Târgu Mureș (Approval No. 2709/27.12.2020).

Contact Information
For questions regarding this study, please contact:
Eugen Silviu Bud
Department of Orthodontics
University of Medicine, Pharmacy, Science and Technology "George Emil Palade" of Târgu Mureș
Email: eugen.bud@umfst.ro
Telephone: ___________________

Consent Statement
I confirm that I have read and understood the information provided above. I voluntarily agree for my child to participate in this study.
Child's Name: ___________________________
Parent / Legal Guardian Name: ___________________________
Relationship to Child: ____________________
Signature: _______________________________
Date: ___________________________________

CHILD ASSENT FORM

Study Title: Correlations Between Cranial Base Morphology and Maxillofacial Development in Growing Patients with Skeletal Deep Bite Malocclusion (Angle Class II Division 2)

Why are we doing this study?

We are doing a study to learn how children's jaws and teeth grow and develop. This may help doctors better understand bite problems and improve orthodontic treatments for children in the future.

What will happen if you agree?

If you say "yes":

- We will look at X-ray images of your teeth and jaws that were already taken as part of your normal orthodontic care.
- We will use information from your dental records to study how your face and jaws grow.

You will NOT need any extra X-rays, tests, or procedures for this study.

Will this hurt or be uncomfortable?

No. We will only use records that already exist from your regular orthodontic visits.

Do you have to take part?

No. It is your choice.

If you do not want to take part, nothing will change about your dental care.

Even if you say yes now, you can change your mind later.

Privacy

Your name will not appear anywhere in the study. Doctors will keep your information private.

If you have questions

You can ask your parents, your doctor, or the researchers any questions at any time.

Assent Statement

I have read (or had explained to me) the information above. I understand what this study is about, and I agree to take part.

Child's Name: ___________________________

Signature: ______________________________

Date: __________________________________
